# Supplementary material for: Prediction of fine-tuned promoter activity from DNA sequence
Source: F1000Res. 2016 Feb 11;5:158. [Version 1] doi: 10.12688/f1000research.7485.1 (PMC4916984; doi:10.12688/f1000research.7485.1)
Supplement: Peer review history

# Peer review: Referee response for version 1

**DOI:** 10.5256/f1000research.8064.r14225

**Published:** 2016-06-20

**Authors:** Jan Grau

**Affiliations:** 1 Institute of Computer Science, Martin Luther University of Halle-Wittenberg, Halle, Germany

The authors present FIrST, an approach for predicting promoter activity from sequence, which won one of the DREAM6 challenges. FIrST is using only simple sequence features in a limited range (100 bp) upstream of the translation start site for making its predictions, which distinguishes it from several other approaches in this field.

 Prediction results are convincing and the method appears to be sound. However, currently the method is not described detailed enough. In addition, I have a few further major and minor concerns regarding the current version of the manuscript:

 Major comments:

1. In the list of features described in section "Feature extraction", some seem redundant to me. For instance, the trinucleotide parameters for bendability are just computed from the k-mers for k=3. Also nucleosome binding prediction was based on trinucleotide preference. Please explain why it may be useful to also include those 3-mer-derived features in addition to the 3-mers themselves.
2. The description of methods in section "Machine learning model exploration" is too coarse. Please provide more detail on the SVMs, linear regression, and regression trees employed. It also remains unclear if the scales of features are normalized somehow, before their values are provided to the SVM.
3. No details are given on the selected 3-mers and 4-mers (Table 1). Please provide a list of the specific k-mers selected by FIrST. It may also be reasonable to discuss potential biological reasons for their importance (as partly covered for TATA-boxes on page 6).
4. Considering Fig. 3, I wondered if the difference in deformability may be related to transcription initiation. Or, stated differently, might we observe an ever clearer signal if all sequences (and their deformability profiles) would be aligned by the transcription start site (TSS) instead of the TrSS? One idea in the same direction, which could contribute to the novelty of the manuscript, would be to evaluate similar profiles (of sequences aligned to TSS or TrSS) for all features found to be informative by FIrST. For instance, one could expect to see something like general fluctuations of G/C content, or the TATA-box in 4-mer profiles as a spike approx. 35 bp before the TSS. From my perspective, this might improve the novelty of the manuscripts and the interpretation of features.

Minor comments:

1. The data from the DREAM6 challenge only consider a special subset of genes (ribosomal genes) and only in yeast. It is unclear if the features derived by the authors' method would also be informative for higher eukaryotes. I understand that this question cannot be finally answered from the DREAM6 data, but the authors might comment on this issue.
2. Figure 1B remains a bit unclear. In the caption and the main text, the authors explain that they use non-overlapping 100bp sub-sequences. However, from the figure it rather seems that they consider upstream sequences of 300 bp, 200 bp and 100 bp (and the full promoter sequence) relative to the translation start site. Please clarify.
3. In section "DREAM6 challenge data" of "Methods", the authors refer to "the sequence 1200 bp upstream of a gene", where "upstream of the translation start site" (as in the remainder of the text) would be more specific.
4. In section "Feature extraction", the authors explain that "each promoter sequence was divided into 100 bp non-overlapping windows", while in the previous section they explain that the full 1200 bp sequences do not extend over the nearest gene. From my understanding, this may result in some of the sequences being shorter than 1200 bp, and their length might not be dividable by 100. Please explain how such cases are handled.
5. At the end of section "Validation of model by DREAM6 consortium", the authors explain that "the overall score was defined as the product of the four P-values", whereas later they explain that -log _10_ of the geometric mean of the p-values was used as the overall measure. Although bot definition are equivalent with respect to the resulting ranking, I would suggest to provide one consistent definition of the overall score.
6. From the manuscript it did not become fully clear if the TA-tracts (also termed poly(dA-dT) tracts in some parts of the manuscript) are tracts of poly "A or T" or tracts of poly "AT"-dinucleotides.
7. In section "Error profile of SVM promoter activity model", the authors explain that natural promoters had (slightly) lower activity than synthetic promoters and that the prediction error of the SVM is lower for natural promoters. However, I did not get the idea, why this should explain that low activity genes had larger prediction errors.
8. In section "Error profile of SVM promoter activity model", the authors explain that one reason why FIrST did not perform well for synthetic promoters is that most mutations had been introduced outside the 100 bp range considered by FIrST. However, this reasoning partly contradicts the claim of the authors that most of the transcriptional activity may be explained from the sequence in that 100 bp window. If this would truly be the case, mutations outside this range should have only minor effects.
9. In the Discussion, the authors mention that TF binding motifs are 6 to 8 bp in length. While this may be true for several yeast TFs, it is not correct for eukaryotes in general and motifs may be wider than 10 bp.

I have read this submission. I believe that I have an appropriate level of expertise to confirm that it is of an acceptable scientific standard, however I have significant reservations, as outlined above.

# Peer review: Referee response for version 1

**DOI:** 10.5256/f1000research.8064.r12380

**Published:** 2016-04-07

**Authors:** Jianhua Ruan

**Affiliations:** 1 Department of Computer Science, The University of Texas at San Antonio, San Antonio, TX, USA

This article describes the winning method of the DREAM6 promoter activity prediction challenge. While a meta analysis of the competing methods participated in the challenge has already been published (Meyer *et al.*, 2013), this article provides more details of the winning method and some additional analysis of the predictive model, which may lead to better understanding of the predictability of gene transcription. While its contribution is undoubtable, this article should be revised to address several issues:

Major issues:

1. The 23 features utilized by the SVM model (as well as their coefficients in the model) is not provided explicitly in the main text nor in the supplement file. Table 1 in the main text shows that 6 trinucleotides and 12 tetranucleotides are important features, but it is nowhere to be found which tri- and tetra-nucleotides they are. For lengths of T or TA-tracts, the supplement file shows several different values, including mean, median and stdev. It is unclear which one is actually used by the SVM model. Similarly, supplement file shows 79 values for deformability and it is unknown which one is used. 
2. In the case of ranking the features by their SVM coefficients, the authors need to clarify if the feature values were normalized prior to model building, as these features are on very different scales and if not normalized the ranking of the coefficients are not very meaningful.
3. The main conclusion in the subsection "Error profile of SVM promoter activity model" do not seem to make sense. First, promoters of low activity had larger prediction error. Then the authors stated that natural promoters had lower activity. This seems to contradict with their observation that the prediction error was significantly less for natural promoters than for mutated promoters.

Minor issues:

1. Authors only mentioned that feature selection was done in WEKA with wrapper. More details need to be given. For example, what was the selection strategy used by the wrapper, e.g., exhaustive search, greedy forward search, backward search, or other types of heuristics? 
2. What is the purpose of first training 1000 SVM classifiers using 66% of data as training and 34% as testing, and then another 500 SVM classifiers using 80% as training and 20% as testing?

I have read this submission. I believe that I have an appropriate level of expertise to confirm that it is of an acceptable scientific standard, however I have significant reservations, as outlined above.

## References

[1] MeyerPSiwoGZeeviDSharonENorelRSegalEStolovitzkyG: Inferring gene expression from ribosomal promoter sequences, a crowdsourcing approach. *Genome Res*.2013;23(11) : 10.1101/gr.157420.113 1928-37 10.1101/gr.157420.113 23950146PMC3814892

# Peer review: Referee response for version 1

**DOI:** 10.5256/f1000research.8064.r12530

**Published:** 2016-03-02

**Authors:** Paul Pavlidis

**Affiliations:** 1 Centre for High-Throughput Biology and Department of Psychiatry, University of British Columbia, Vancouver, BC, Canada

Siwo *et al*. give a detailed report of their entry to the DREAM promoter activity prediction assessment, conducted in 2011. The paper describing the results of the assessment appeared in 2013 (Meyer *et al*.), and the entry from Siwo *et al*. (“FiRST”) was the top-performer overall. Meyer *et al*. gives few details about the specific methods, mentioning only that the FiRST entry used an SVM and did not use TF binding site motif information. Here it is clarified that FiRST is a simple method that uses only part of the sequence and the most prominent features were about nucleotide content.

Because it is perhaps a little eye-opening (even embarrassing, depending on one’s point of view) that the best method in the assessment is so simple, this paper is an important footnote to Meyer *et al*. but it could be fleshed out further to get at what is going on. My suggestions for revisions are to give more detail about the properties of the sequences used and the relationship to performance.

FiRST predicts from only the 100 bases of sequence upstream of the translation start (which was considered as part of the promoter by DREAM; I note this is not “upstream of the gene” as described by Siwo *et al*. in the methods section), and that their predictions were dominated by the effect of a simple measure of G content. Siwo *et al*. report that they did worse at predicting the synthetically mutated promoters (this was apparently not true overall across methods as reported by Meyer *et al*.). In Meyer *et al*., adding tf binding information to FiRST improved performance.

The authors mention this, but the most important reason that FiRST does poorly at predicting the synthetic mutations seems to be that most of the mutations (seems to be 29 out of 33, based on Table 1 of Meyer *et al*.) are not in the 100 bp window used. That is, because in most cases these synthetic sequences were (as I understand it) identical in features to other examples while having different activities, for the purposes of FiRST, they could only introduce prediction errors. In light of this fact the rest of the speculation about why performance varied in this way seems extraneous.

It would also be useful to see more detailed information on the sequences used (e.g., the G content or other features), and the prediction error in each case. How well does one predict using G content alone? This might all be reconstructed from the data supplement helpfully provided, but the authors should consider providing the analysis. It also seems reasonable to ask for more details about the performance of other sequence windows.

The main other missing piece from this paper is any discussion or evidence that the method works beyond the narrow confines of the DREAM setup. Even for the RP genes, does it make a useful prediction, that increasing the G content of RP promoters in that 100 bp window will decrease promoter activity? I am fine with leaving this as “future work” but it would be worth mentioning.

Figure 2B is apparently the same as part of Figure 1E from Meyer *et al*., except FiRST is not marked (actually there is a small difference in the values plotted; the combined score for FiRST looks closer to 2 than the 1.87 reported and plotted in Meyer *et al*.). The authors should clearly cite Meyer *et al*. in the figure caption as the source of the data for this figure, or simply point the readers to Meyer *et al*., or else explain where the data came from if not from Meyer *et al*.

I have read this submission. I believe that I have an appropriate level of expertise to confirm that it is of an acceptable scientific standard.
